# A stress fracture of the base of the acromion: a case report

**DOI:** 10.1186/1471-2474-15-302

**Published:** 2014-09-12

**Authors:** Eduardo Angeli Malavolta, Jorge Henrique Assunção, Edwin Eiji Sunada, Mauro Emilio Conforto Gracitelli, Arnaldo Amado Ferreira Neto

**Affiliations:** Shoulder and Elbow Group, Institute of Orthopedics and Traumatology, School of Medicine, University of São Paulo (Universidade de São Paulo; USP), Address: Rua Ovidio Pires de Campos, 333 São Paulo, SP Brazil

**Keywords:** Scapula, Fractures, Stress, Fracture fixation, Pseudarthrosis

## Abstract

**Background:**

Stress fractures of the base of the acromion are rare and tend to progress well when conservatively treated. The need for surgery due to this type of fracture has only been reported in two case reports.

**Case presentation:**

A 39-year-old patient, manual laborer, with a stress fracture at the base of the acromion that required surgical treatment due to persistent symptoms and consolidation failure.

**Conclusion:**

We described a new mechanism of injury for stress fractures of the base of the acromion. After the failure of conservative treatment, the patient exhibited good results with osteosynthesis with a plate and screws, with no need for a bone graft.

**Electronic supplementary material:**

The online version of this article (doi:10.1186/1471-2474-15-302) contains supplementary material, which is available to authorized users.

## Background

Acromion fractures of the scapula are rare and have been given little consideration since they have traditionally been managed nonoperatively, often with favorable outcomes[1]. Indications for operative management include symptomatic nonunion, displaced fractures, or acromion fractures associated with other lesions of the superior shoulder suspensory complex[1, 2]. Fractures may be caused by a direct lateral blow to the shoulder, avulsed from indirect forces to the deltoid muscle, or the result of overuse injuries[1].

Stress fractures of the base of the acromion[3–10] are very rare and are usually successfully treated using conservative procedures[4, 5, 7, 8]. The need for surgery due to this type of fracture has only been reported in two case reports [9, 10]. We report a case of a patient with a stress fracture of the base of the acromion who underwent surgery because of a symptomatic nonunion.

## Case presentation

A 39-year-old right hand dominant male sought our services with a history of eight months of pain in the posterior region of the left shoulder, with no history of acute trauma. The patient stated that the pain began after starting a job where he carried sandbags of approximately 25 kg on the affected shoulder. The pain began after two months on the new job and progressively worsened with time. The pain was worse with exertion. The pain was partially relieved with rest and the use of non-steroidal anti-inflammatory drugs. At the time of his first appointment, the patient was unable to work, and he did not report any comorbidity.

**Figure 1:**
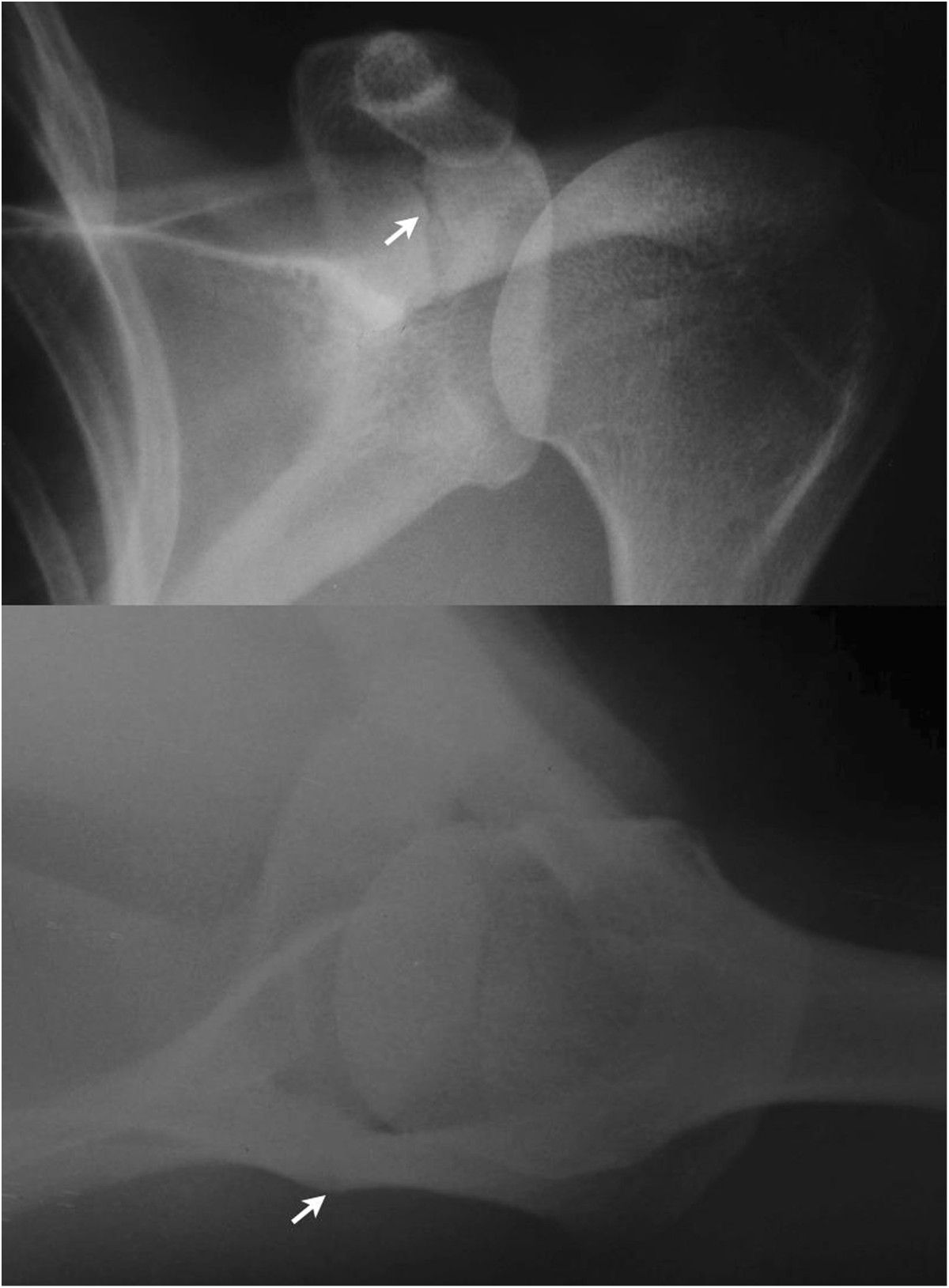
**Preoperative radiographs.** Radiographs in the anteroposterior and axillary views demonstrating (arrows) the transverse fracture and the lack of deviation of the base of the acromion.

**Figure 2:**
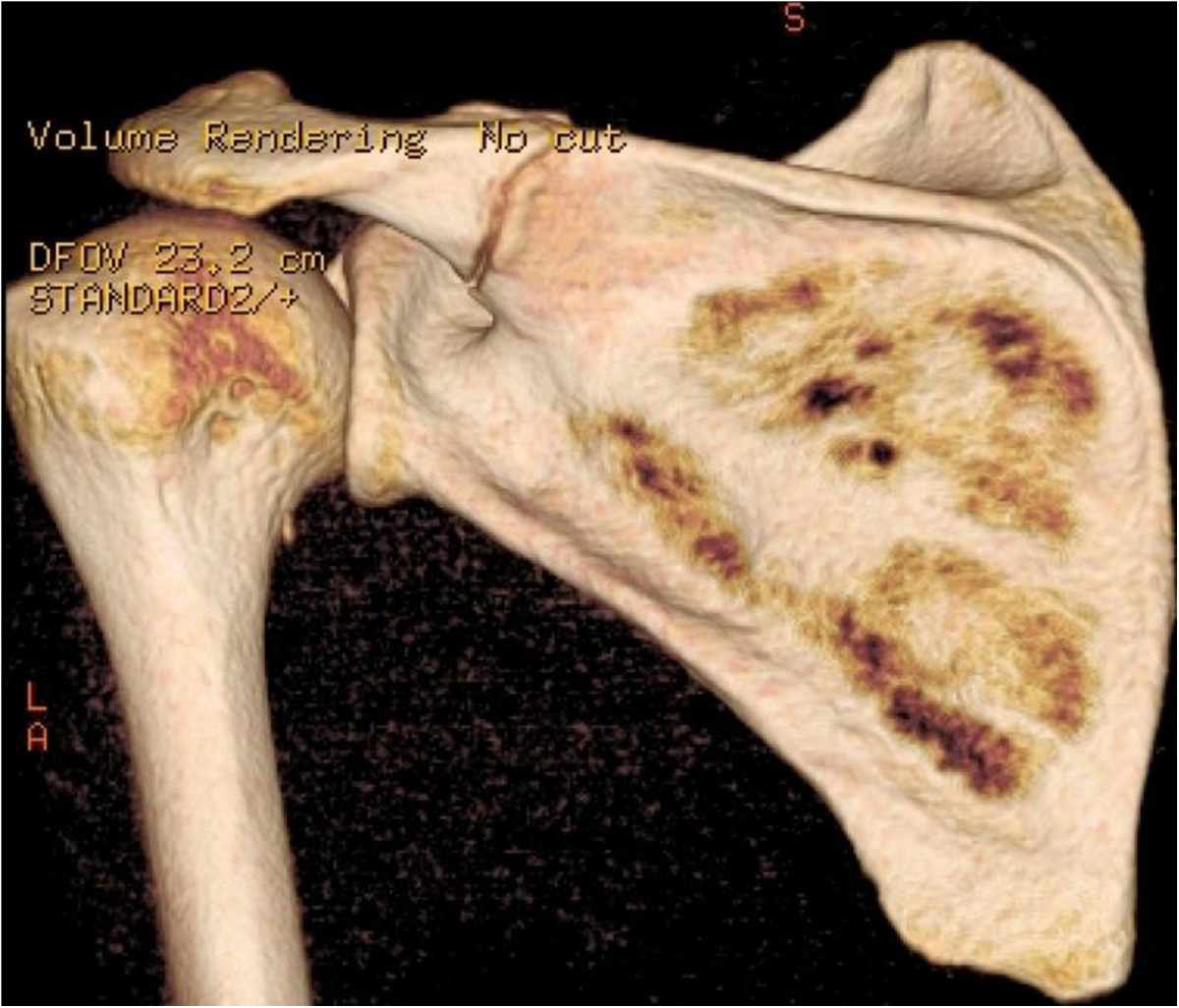
**Preoperative CT.** Posterior view of the left shoulder on CT with 3-dimensional reconstruction performed after 4 months of conservative treatment. An absence of consolidation of the fracture at the base of the acromion on the spinoglenoid notch can be observed.

**Figure 3:**
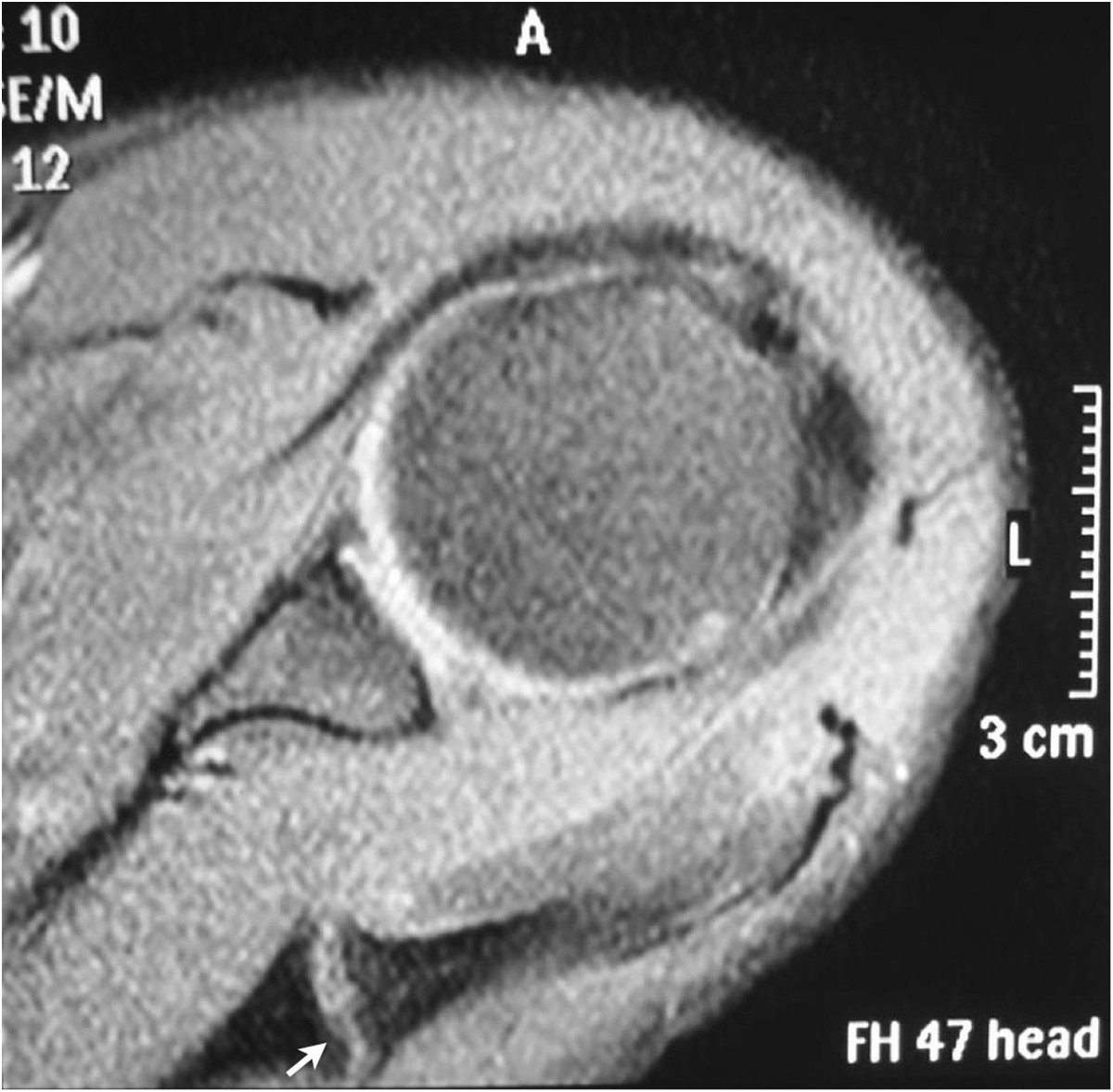
**Preoperative magnetic resonance imaging (MRI).** An axial section of the T2-weighted MRI demonstrating a hypertrophic pseudarthrosis of the fracture of the base of the acromion (arrow).

Physical examination of the left shoulder revealed no gross deformity. On palpation, the patient reported pain over the scapular spine, approximately 5 cm medial to the lateral border of the acromion. The passive range of motion was normal, and active forward elevation, abduction and external rotation were limited to 130°, 90° and 30°, respectively. The patient exhibited pain and decreased strength on the Jobe and infraspinatus tests as well as negative signs for a superior labrum anterior and posterior (SLAP) lesion and acromioclavicular arthrosis. The functional assessment scored 10 points on the University of California at Los Angeles (UCLA) Shoulder Rating scale and 7 points on the visual analog scale.The initial radiographs revealed a transverse fracture of the base of the acromion on the spinoglenoid notch without deviation (Figure 1). A non-surgical treatment with analgesics, rest, absence from work, and physical therapy (analgesia, stretching, and strengthening) was used for four months, with no clinical improvement. New imaging exams (radiography, computed tomography (CT), and magnetic resonance imaging (MRI)) revealed the absence of consolidation (Figures 2 and 3). Thus, after 4 months of conservative treatment and 1 year after the onset of symptoms, surgical treatment was recommended.

**Figure 4:**
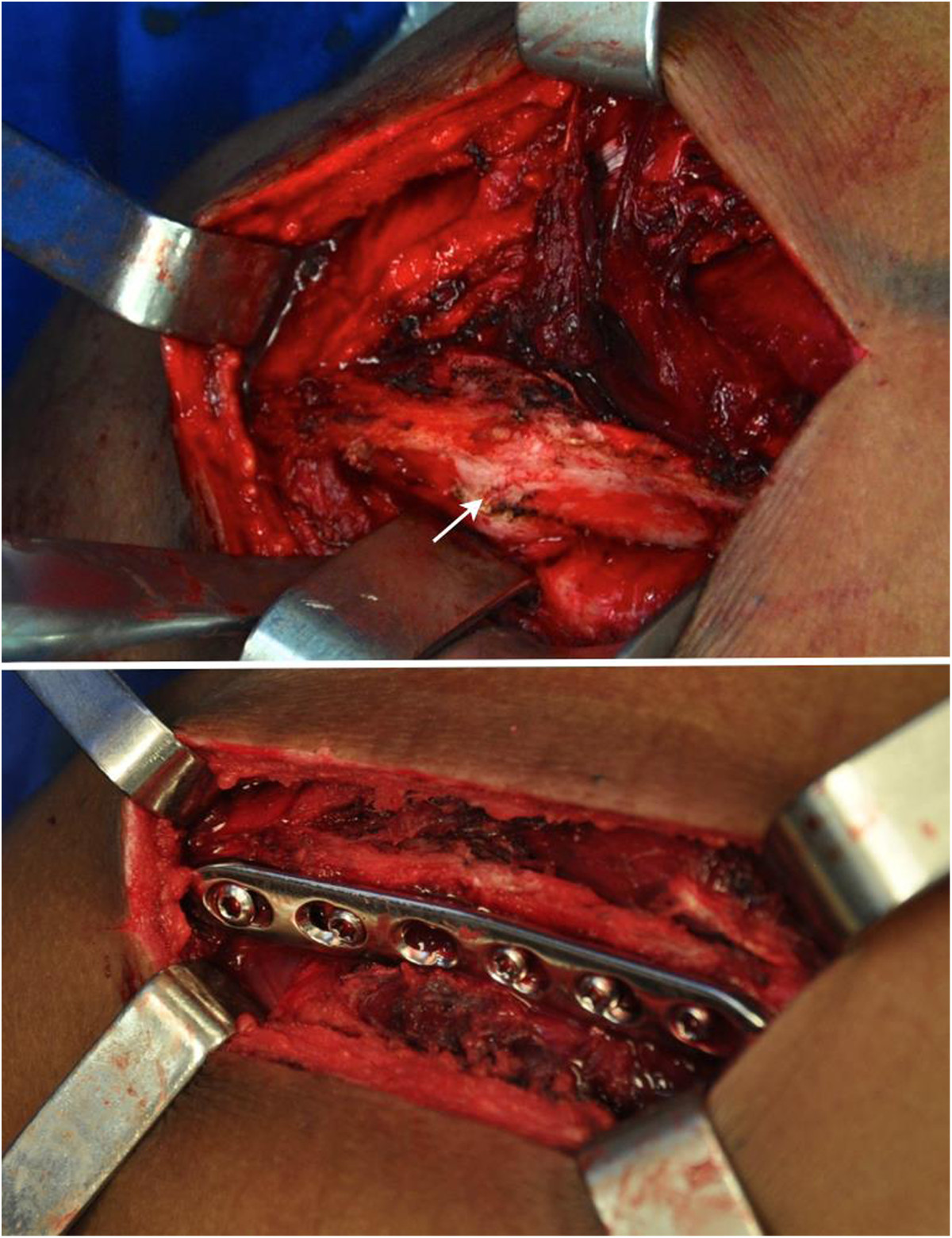
**Intraoperative images.** The arrow indicates the location of the pseudarthrosis focus after partial resection of the hypertrophic callus and after the scapular spine was placed in the normal position. Below, the fracture was fixed with a 3.5 mm LCP with 3 screws proximal to the focus and 2 screws distal to the focus.

**Figure 5:**
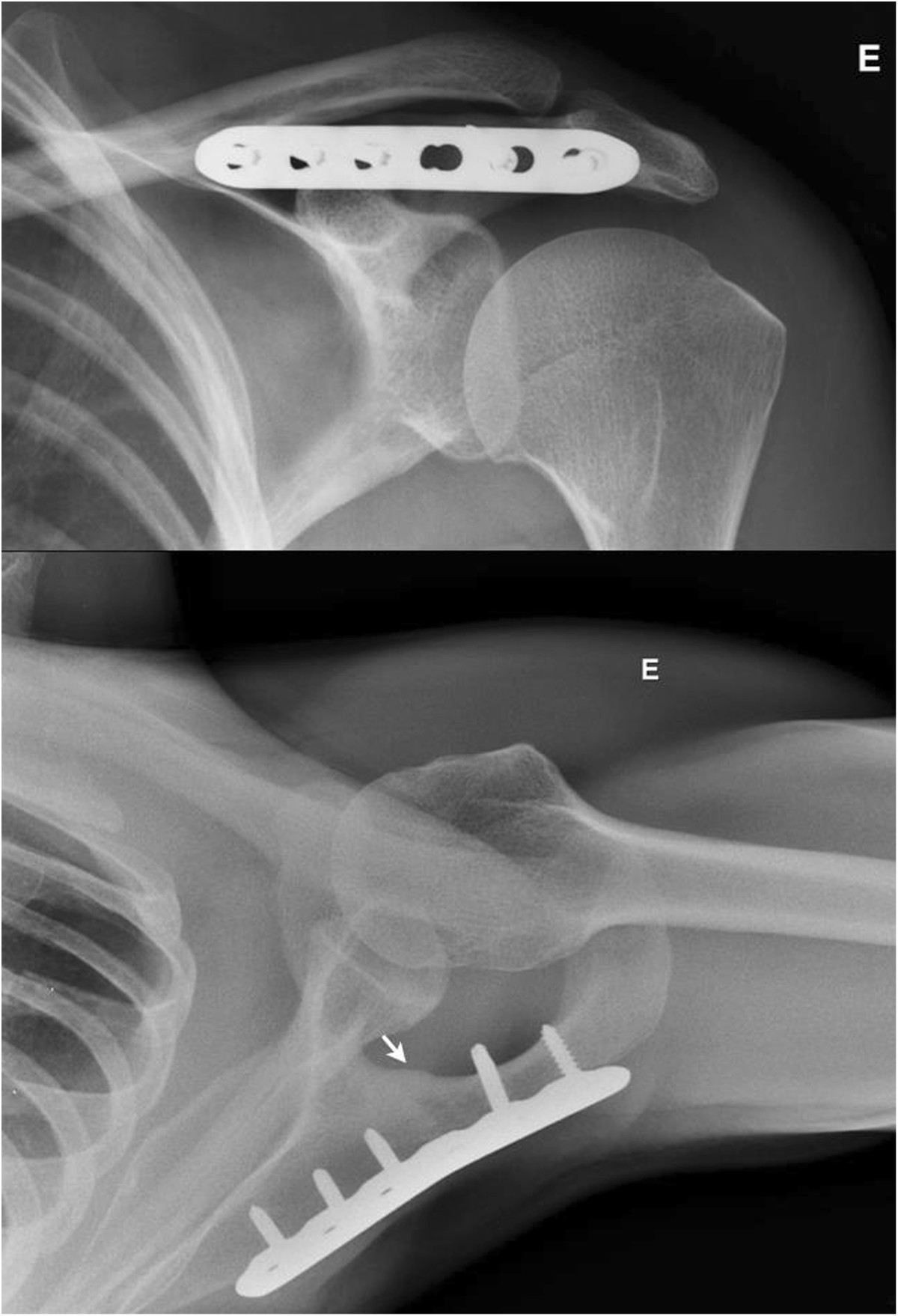
**Postoperative radiographs.** Postoperative anteroposterior and axillary radiographs demonstrating fracture consolidation (arrow).

**Figure 6:**
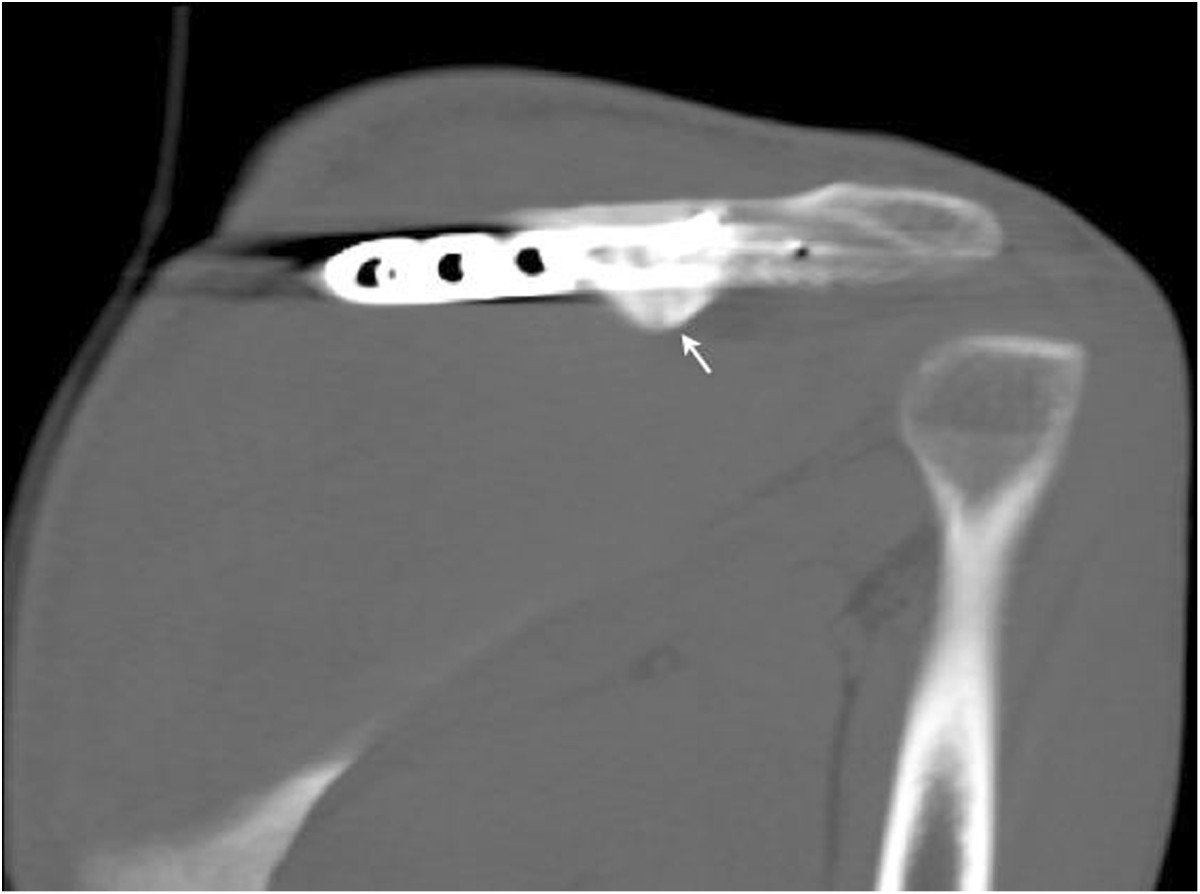
**Postoperative CT.** Oblique coronal reconstruction CT images of the left shoulder performed 5 months after the surgery demonstrating fracture consolidation.

The patient was placed in the prone position, and a 10 cm incision was made along the scapular spine towards the lateral edge of the acromion. The trapezius-deltoid fascia was opened towards the spine. The deltoid and trapezius muscles were detached, exposing the pseudarthrosis focus, with hypertrophic callus. A partial resection of the hypertrophic callus was performed, and the scapular spine was anatomically reduced. The osteosynthesis was performed with a 6-hole 3.5-mm locking compression plate (LCP) with 3 screws locked proximally and 2 distal screws, 1 of them locked and 1 cortical screw placed eccentrically, resulting in an interfragmentary compression of 1 mm (Figure 4). The lateral fixation with 3 screws was not possible due to the location of the fracture. No bone graft was used, and the incision was closed in layers, reinserting the muscles using transosseous sutures.The operated limb was immobilized with an arm sling for 6 weeks, and the mobilization of the hand, wrist, and elbow was performed beginning on the 1st postoperative day. Passive shoulder movements were started at 3 weeks, free active movements at 6 weeks and resistance movements at 8 weeks. After 4 postoperative months, the patient had recovered full range of motion. The radiograph (Figure 5) and the CT performed at 5 postoperative months (Figure 6) revealed the healed fracture. After 6 months, the patient rarely reported mild pain and returned to his work. The UCLA scale was 33 at 6 months of follow-up and 35 at 12 months. The maximum UCLA score is 35 points. The patient recovered full painless range of motion and normal strenght.

## Conclusion

Stress fractures of the base of the acromion are rare [3–10], and the literature only describes case reports. Possible causes for the occurrence of these fractures are rotator cuff arthropathy [3, 6], history of reverse arthroplasty [10], manual lifting work [5], golfing [4, 7], football [8], and artistic gymnastics [9]. Our report describes the act of repeatedly carrying weight on the shoulder as a new mechanism of injury for this condition.

Conservative treatment is considered a viable treatment option in most reports [3–7, 10]. In patients with low functional demands, even when there is no consolidation, this treatment can be effective in improving symptoms [6]. However, osteosynthesis is necessary when the symptoms persist [9, 10]. Our patient had been complaining for 12 months at the time of surgery, with no potential for consolidation according to our assessment.

Similar to Wahlquist et al. [10], we believe that fractures of the base of the acromion exhibit different characteristics than fractures of the acromion and the spine. Due to the insertion of the deltoid and trapezius muscles, these fractures exhibit a greater tendency towards instability and may justify a change in muscle function with functional impairment and the increased risk of pseudarthrosis.

The surgical techniques described for the osteosynthesis of these fractures involve plates and screws [10] or the use of tension-band wiring using autologous [9] or synthetic bone grafts [10]. Our report differs from previous reports in that we did not use a bone graft. We believe that the reason for nonunion of the fracture was the lack of stability and not a vascularization failure. There was no bone loss and fracture was a hypertrophic nonunion.

It is noteworthy that there is an inconsistency in the nomenclature for this type of fracture in the literature. Even when occurring in the same anatomical region, next to the spinoglenoid notch, these fractures are treated as fractures of the acromion [3, 5, 7, 9], of the base of the acromion [6, 8, 10], or of the spine [11]. The previous reports could be used the classification of Ogawa and Naniwa [12]. These authors advocate that acromion fractures should be classified into two types in terms of clinical consideration: type I fractures, comprising those of the anatomic acromion and the extremely lateral scapular spine, and type II fractures, located in the more medial spine and descending to the spinoglenoidal notch. Our patient had a type II fracture.

Ultimately, we described a new mechanism of injury for stress fractures of the base of the acromion. After the failure of conservative treatment, the patient exhibited good results with osteosynthesis with a plate and screws, with no need for a bone graft.

### Consent

Written informed consent was obtained from the patient for publication of this case report and any accompanying images. A copy of the written consent is available for review by the Editor of this journal.

## Authors’ information

EAM, EES, MECG and JHA are assistant physicians at Shoulder and Elbow Group of the University of São Paulo. AAFN is MD-PhD, Chair of the Shoulder and Elbow Group.

## Authors’ original submitted files for images

Below are the links to the authors’ original submitted files for images. Authors’ original file for figure 1 Authors’ original file for figure 2 Authors’ original file for figure 3 Authors’ original file for figure 4 Authors’ original file for figure 5 Authors’ original file for figure 6

## Abbreviations

SLAP: Superior Labral Anterior and Posterior
UCLA: University of California at Los Angeles
CT: Computed tomography
MRI: Magnetic resonance imaging
LCP: Locking compression plate.
