# Supplementary figures and images for: A stress fracture of the base of the acromion: a case report
Source: BMC Musculoskelet Disord. 2014 Sep 12;15:302. doi: 10.1186/1471-2474-15-302 (PMC4175635; doi:10.1186/1471-2474-15-302)

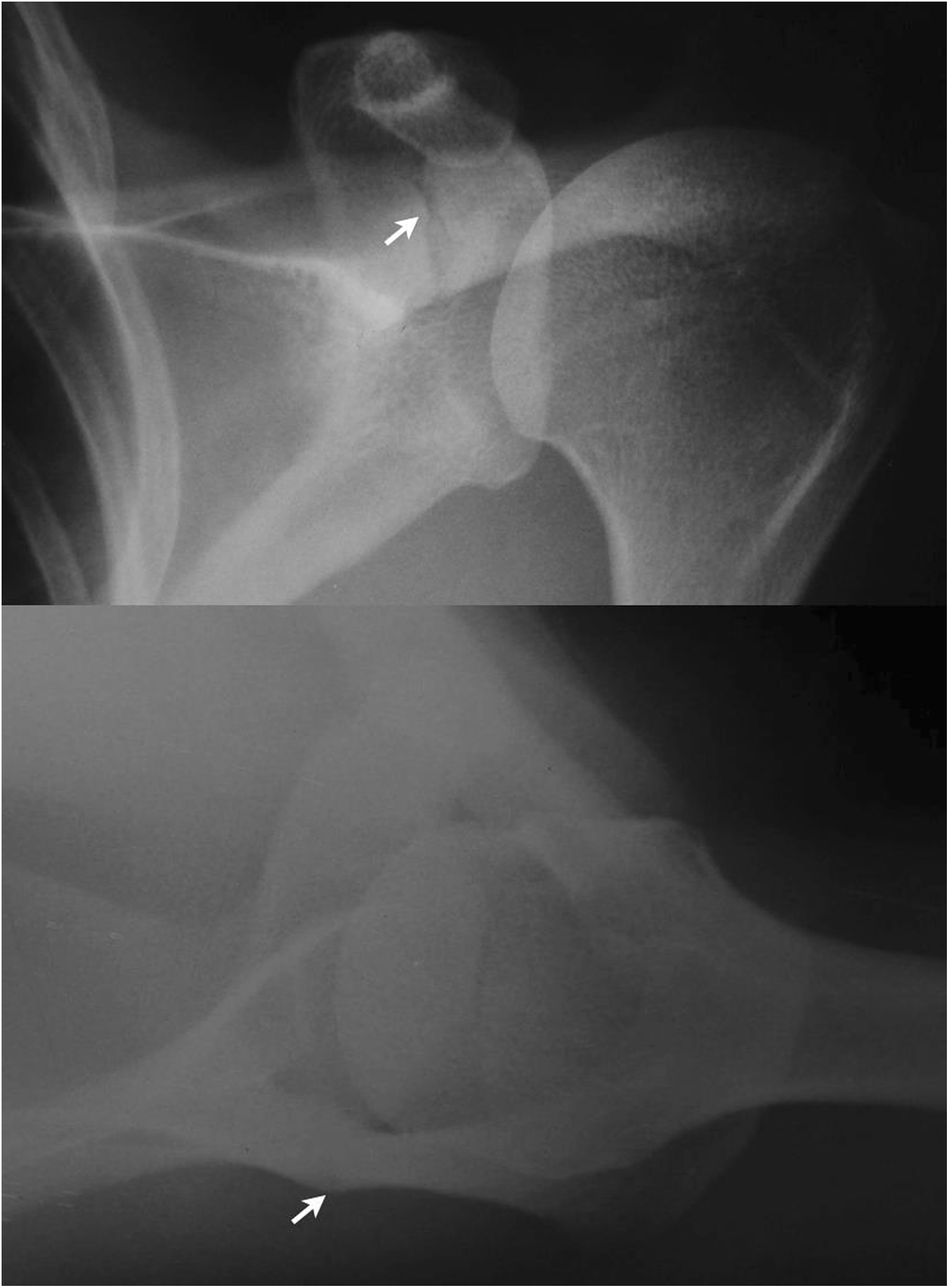

Supplement: Supplementary file 1 — Authors’ original file for figure 1 [file 12891_2013_2246_MOESM1_ESM.tif]

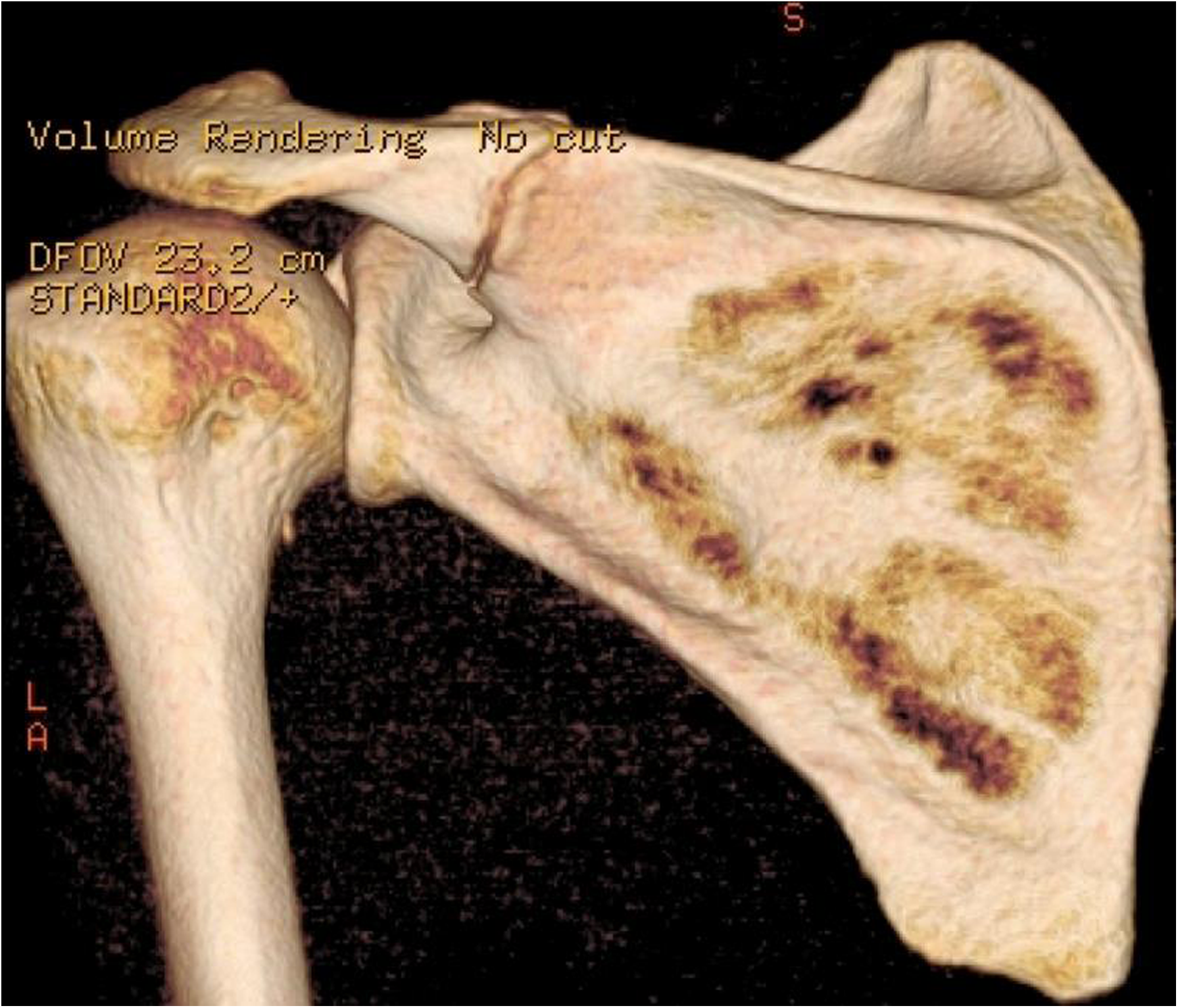

Supplement: Supplementary file 2 — Authors’ original file for figure 2 [file 12891_2013_2246_MOESM2_ESM.tif]

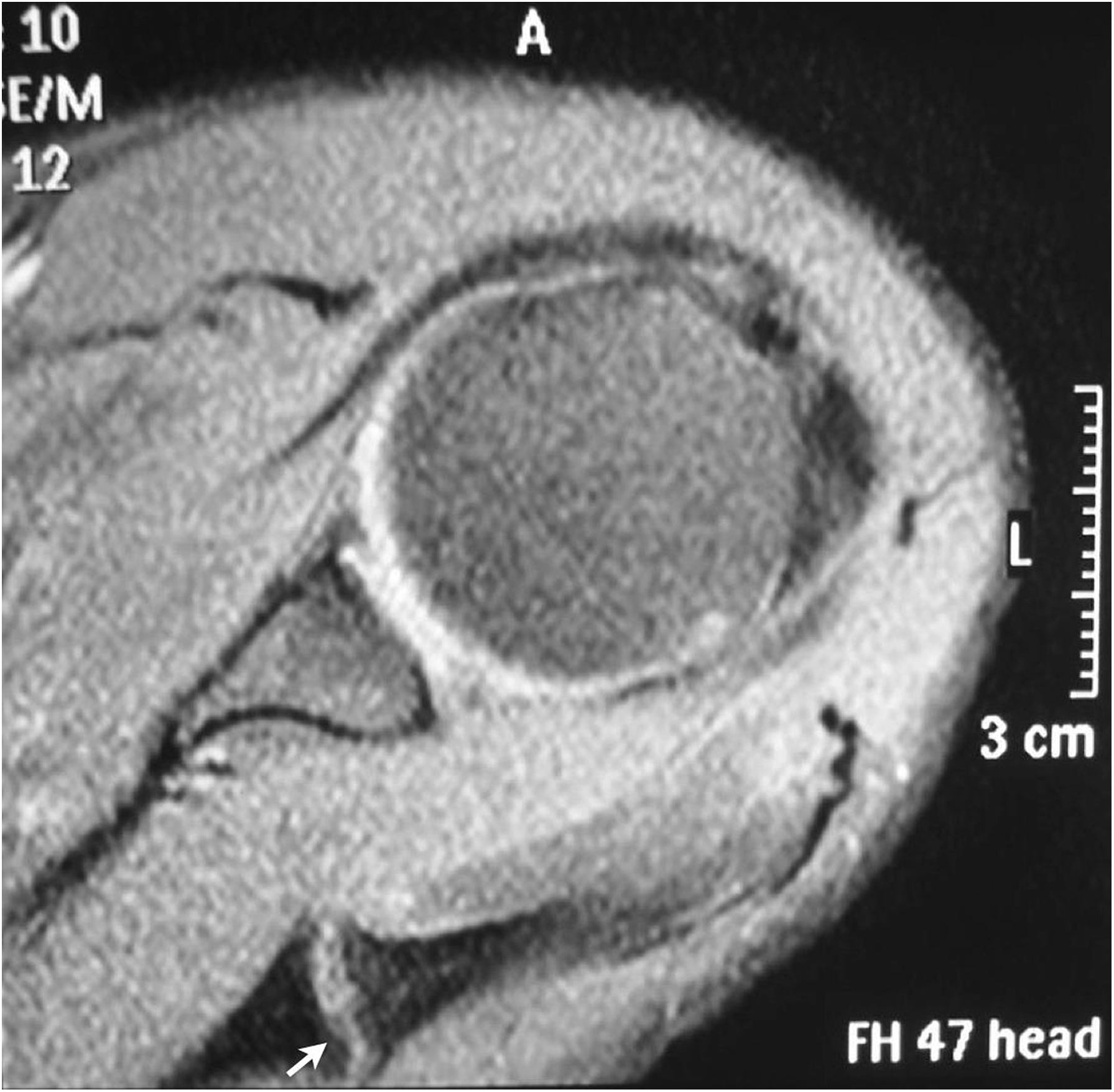

Supplement: Supplementary file 3 — Authors’ original file for figure 3 [file 12891_2013_2246_MOESM3_ESM.tif]

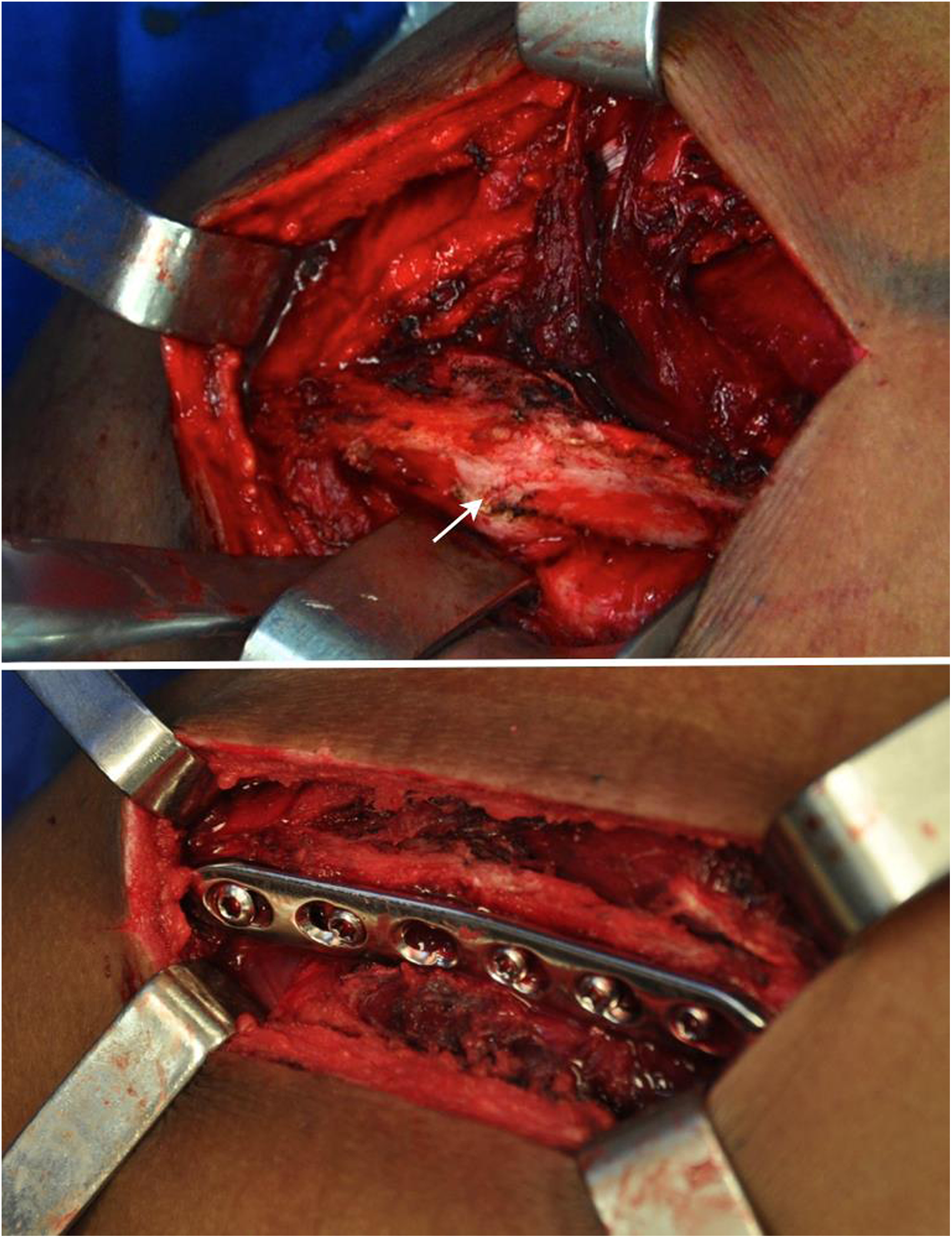

Supplement: Supplementary file 4 — Authors’ original file for figure 4 [file 12891_2013_2246_MOESM4_ESM.tif]

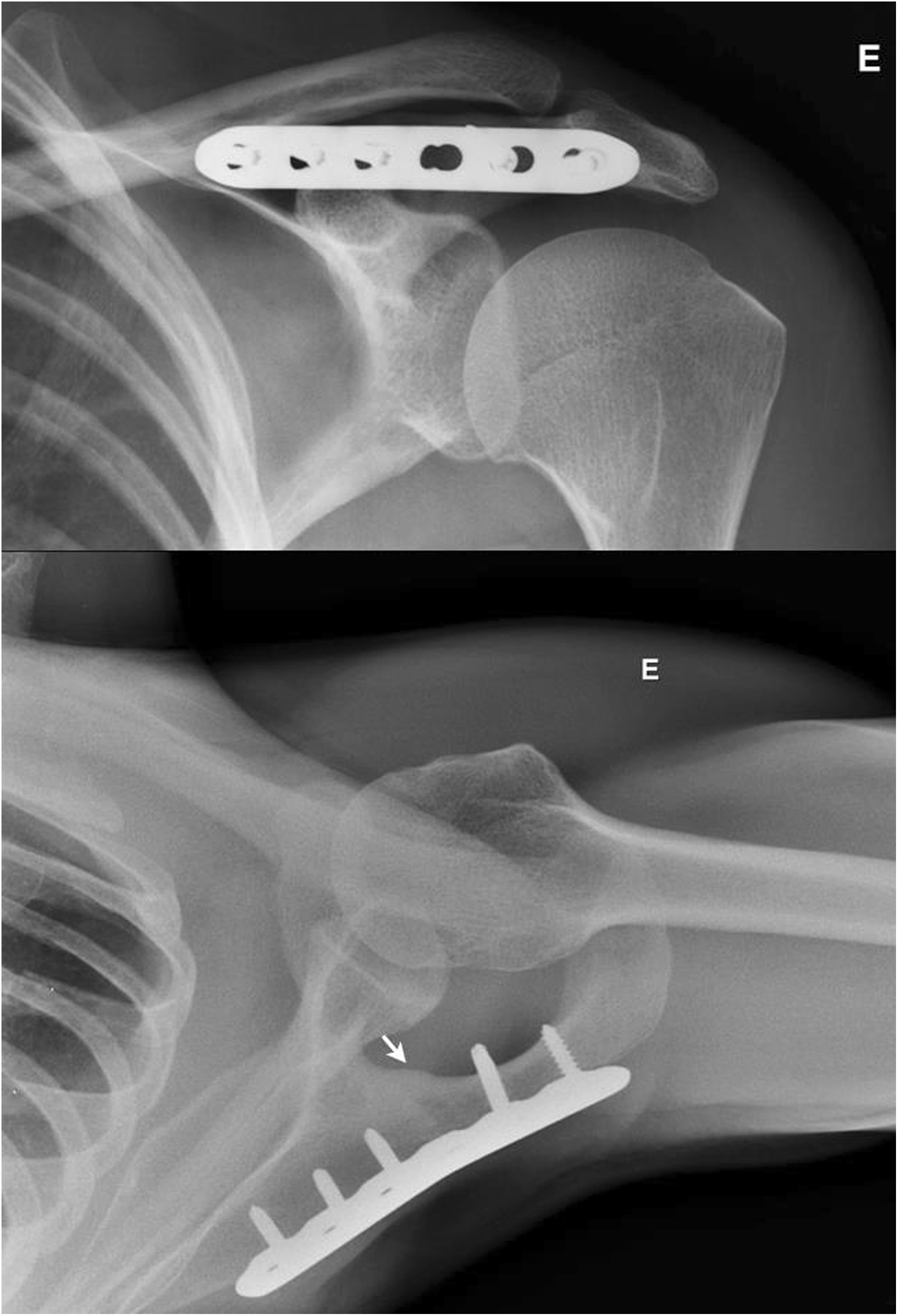

Supplement: Supplementary file 5 — Authors’ original file for figure 5 [file 12891_2013_2246_MOESM5_ESM.tif]

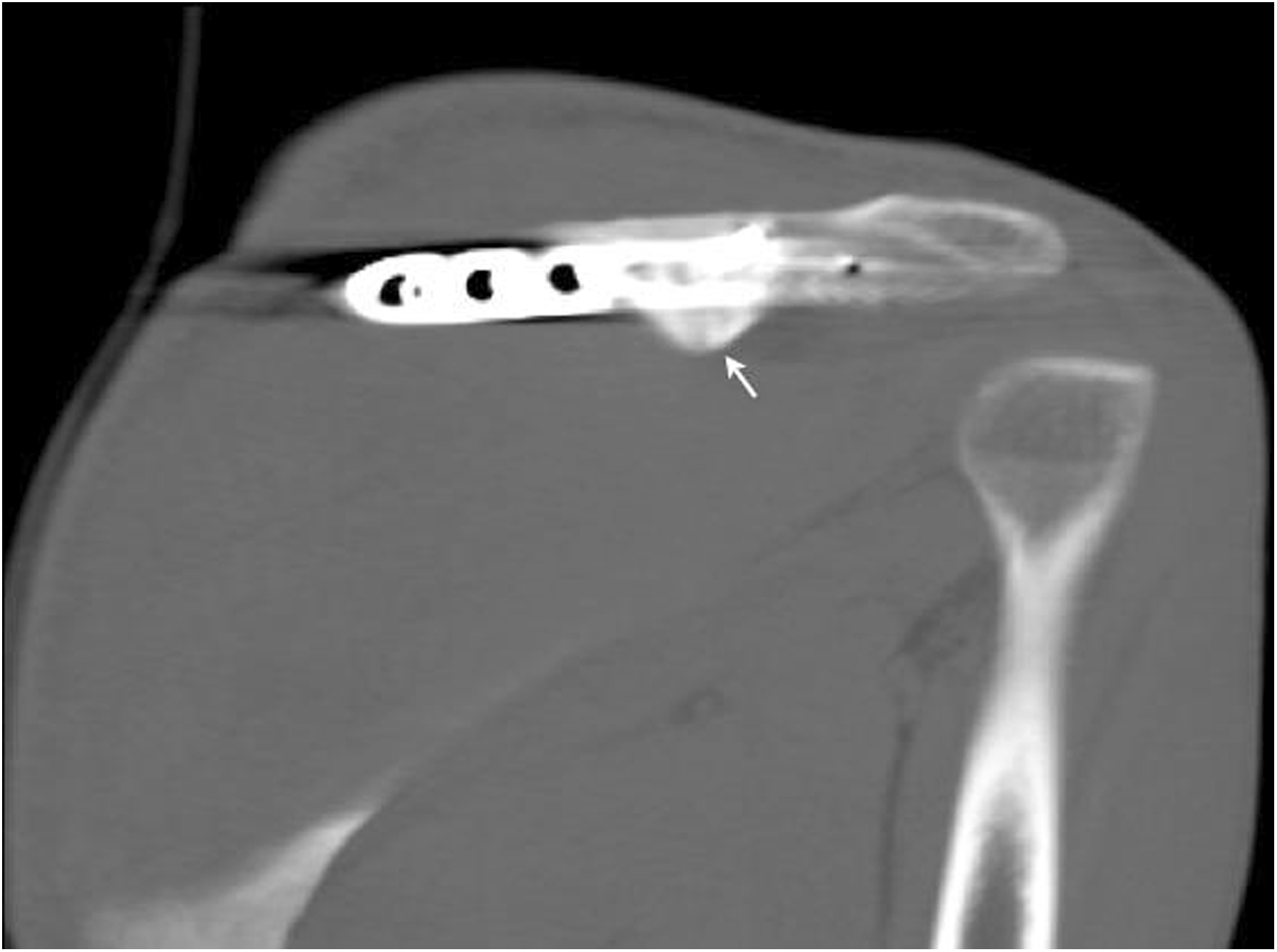

Supplement: Supplementary file 6 — Authors’ original file for figure 6 [file 12891_2013_2246_MOESM6_ESM.tif]
